# A Practical Treatise on Operative Dentistry

**Published:** 1859-12

**Authors:** J. Taft

**Affiliations:** Professor of Operative Dentistry in the Ohio College of Dental Surgery


A Practical Treatise on Operative Dentistry. By J. Taft, Professor of Operative Dentistry in the Ohio College of Dental Surgery. Philadelphia: Lindsay & Blackiston, Publishers.

The progress of Dental Science has been such that it is altogether impracticable to condense into the limits of a single book all that a good student expects to learn and has a right to know from reading ; and hence, text-books devoted to the different departments of the profession, are a necessity of the age. In regard to the general principles of our professional science, very much can be learned by drawing on the literature of our parent, the medical profession ; but when it comes to the practical application of our attainments to the wants of our race, it is evident that we must be self-reliant.

A want was felt by the whole profession, and especially by the younger members of it, in regard to Operative Dentistry. It is true that much had been written, and well written, on the subject ; but as it lay in fragments all over the field of past periodical dental literature, it was not available, without great research. And, as it was necessarily mixed up with much that was common-place, if not useless, the mind of the student was over-taxed, and often, too, much perplexed by the research to discriminate clearly in regard to the merits of what he read. To supply this want, the work under consideration seems to be, in the main, well adapted. It goes over the whole field of Operative Dentistry, giving in detail the various modes of practice which are entitled to consideration.

The work is divided into thirteen chapters, and is so arranged that reference to any subject contained in it is easy.

It is not our purpose to notice at length or minutely, the various positions and doctrines laid down by the author. Long and intimate association has made the writer very familiar with his sentiments ; and while we would not agree with every one of these, it would be somewhat strange if we should disagree with many of them.

The first chapter is mainly introductory, but, withal, highly practical. The second dives right into the great subject, "CARIES OF THE TEETH," giving its general characteristics, its causes, its usual localities, its consequences, and its treatment. In speaking of black decay, the author states that the cause of the dark color is "most probably a metallic oxyd, as iron, sodium, potassium, and calcium are found in the saliva and mucus in several combinations."

This is hardly satisfactory ; for the oxyds of the last named three metals, *soda, potash* and *lime,* are *white,* while the sesquioxyd of iron, the one most likely to be found under the circumstances, is red.

The causes of caries, both predisposing and exciting, are clearly set forth, and its consequences are as faithfully depicted. A few pages on the general treatment of the disease closes the chapter, and brings the reader to the consideration of that most important operation of Dental Surgery, *filling teeth.*

The general principles of this operation are clearly discussed, the materials for filling, both proper and improper, are carefully considered ; and the villainous amalgams are, of course, condemned.

Chapter fourth is devoted to the consideration of instruments for filling; and these are, perhaps, better classified than ever before.

But we have not time or space to notice the work in detail. All the important operations, in all their modifications, are clearly discussed by the author ; and the work is highly practical throughout.

The publishers have surpassed themselves. We have not before seen so neat a text-book. The type, paper, and especially the illustrations, are perfect. W.

